# Knotty is nice: Metabolite binding and RNA-mediated gene regulation by the preQ_1_ riboswitch family

**DOI:** 10.1016/j.jbc.2024.107951

**Published:** 2024-10-30

**Authors:** Daniil Kiliushik, Coleman Goenner, Matthew Law, Griffin M. Schroeder, Yoshita Srivastava, Jermaine L. Jenkins, Joseph E. Wedekind

**Affiliations:** 1 Department of Biochemistry and Biophysics, University of Rochester School of Medicine and Dentistry, Rochester, New York, USA; 2 Center for RNA Biology, University of Rochester School of Medicine and Dentistry, Rochester, New York, USA

**Keywords:** protein translation, transcription regulation, Shine-Dalgarno sequence, pseudoknot, allosteric binding, cooperativity, molecular recognition, queuosine, quintuple base motif

## Abstract

Riboswitches sense specific cellular metabolites, leading to messenger RNA conformational changes that regulate downstream genes. Here, we review the three known prequeosine_1_ (preQ_1_) riboswitch classes, which encompass five gene-regulatory motifs derived from distinct consensus models of folded RNA pseudoknots. Structural and functional analyses reveal multiple gene-regulation strategies ranging from partial occlusion of the ribosome-binding Shine-Dalgarno sequence (SDS), SDS sequestration driven by kinetic or thermodynamic folding pathways, direct preQ_1_ recognition by the SDS, and complete SDS burial with in the riboswitch architecture. Family members can also induce elemental transcriptional pausing, which depends on ligand-mediated pseudoknot formation. Accordingly, preQ_1_ family members provide insight into a wide range of gene-regulatory tactics as well as a diverse repertoire of chemical approaches used to recognize the preQ_1_ metabolite. From a broader perspective, future challenges for the field will include the identification of new riboswitches in mRNAs that do not possess an SDS or those that induce ligand-dependent transcriptional pausing. When choosing an antibacterial target, the field must also consider how well a riboswitch accommodates mutations. Investigation of riboswitches in their natural context will also be critical to elucidate how RNA-mediated gene regulation influences organism fitness, thus providing a firm foundation for antibiotic development.

Most riboswitches are *cis*-acting, RNA-regulatory elements that control gene expression by sensing the cellular levels of one or more cognate ligands (1, 2, 3, 4). Predominantly found in the 5′-leader sequences of bacterial mRNAs, riboswitches fold into structurally conserved aptamer domains that bind directly to specific ligands (5). Recognition occurs when the ligand reaches a threshold concentration within the cell that induces a structural “switch” in the mRNA, thus allosterically changing the conformation of an adjacent expression platform (5, 6). In this manner, the ligand-dependent conformational change alters the accessibility of RNA sequences that control downstream gene expression (7, 8, 9) (Fig. 1*A*). Unlike proteins that incorporate the 20 common amino acids into a selective and chemically diverse binding pocket, riboswitches comprise only the four standard ribonucleotides (10). The relatively short lifespan of riboswitches prevents posttranscriptional nucleotide modifications such as those found in tRNAs or rRNAs (11, 12). As such, riboswitches must gain functionality by adopting elegant three-dimensional folds, albeit with limited chemical diversity relative to proteins (13).

**Figure 1:**
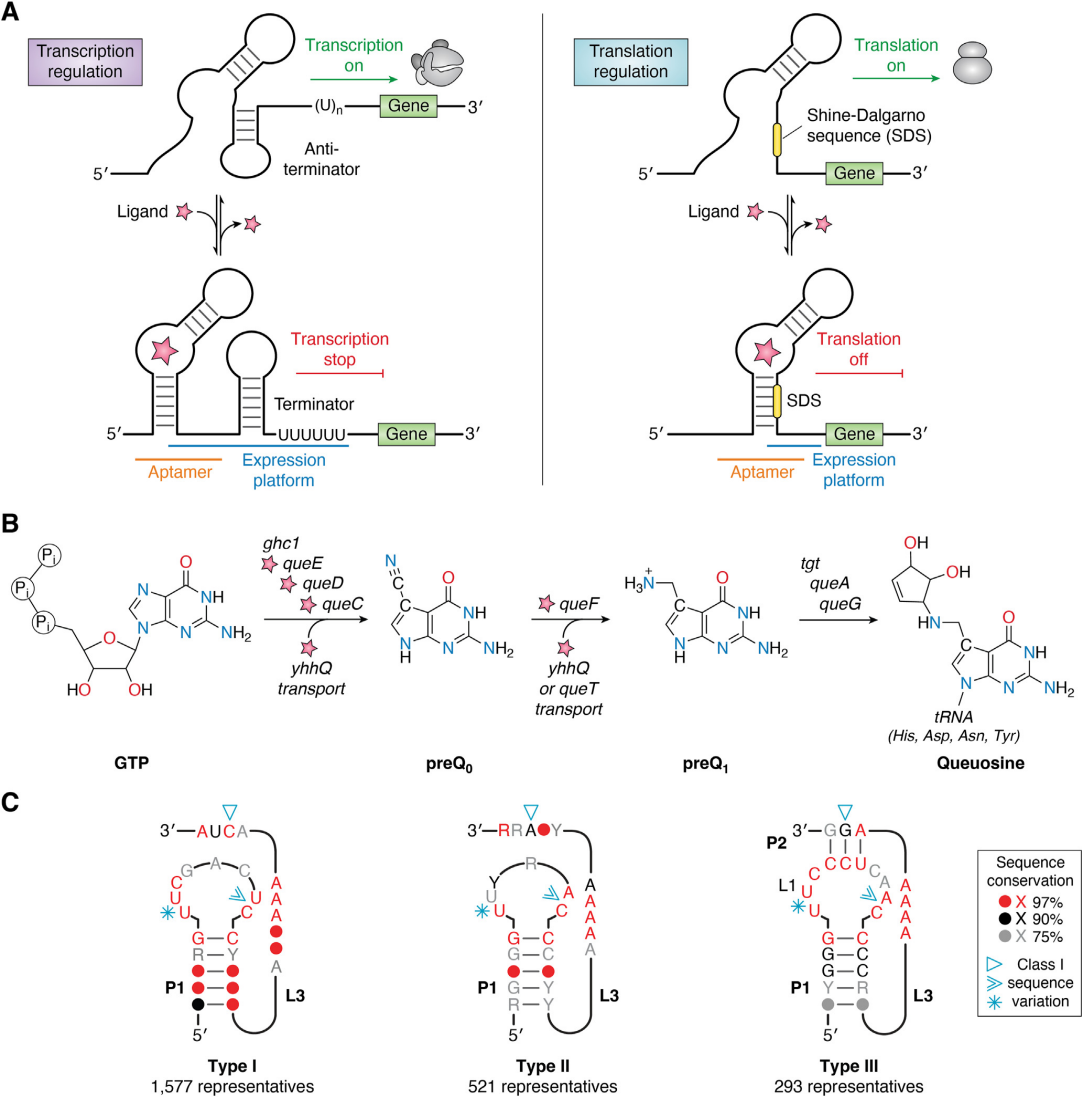
**Gene-regulatory models of bacterial riboswitches, biosynthesis of queuosine and preQ**_**1**_**class I covariation models.***A*, *left panel*: transcription control entails ligand-dependent partitioning between mRNA folds that support or disfavor polymerase activity. In low levels of ligand (*star*), the riboswitch aptamer folds poorly, allowing formation of an antiterminator hairpin that favors transcription. When abundant the ligand supports an alternate fold, resulting in a strong terminator helix followed by a polyuridine tract that disfavors transcription. *Right panel*: translation control entails ligand-dependent partitioning between folds that support or disfavor ribosome access to the Shine-Dalgarno sequence (SDS). In low levels of ligand (*star*), the riboswitch aptamer folds poorly, allowing access to the SDS for translation initiation. When ligand is abundant an alternative fold favors ligand binding, resulting in SDS burial that attenuates translation. *B*, the prokaryotic pathway for *de novo* queuosine biosynthesis begins with GTP, which is converted to preQ_1_ (7-deaza-7-aminomethyl guanine) by the *ghc1* gene product (GTP cyclohydrolase I) and enzymes encoded by the *queCDEF* operon (136). The preQ_1_ precursor is preQ_0_, which contains a nitrile group that is reduced by QueF in the Q pathway (137). However, preQ_0_ also serves as a scaffold for other natural products such as toyocamycin and sangivamycin, which have antibacterial properties (136). Bacteria can also salvage preQ_0_ and preQ_1_ using transporters encoded by *queT, yhhQ*, and other genes (119, 120, 138). PreQ_1_-sensing riboswitches have been found to regulate the biosynthetic *queCDEF* operon, as well as *queT* and *yhhQ* (69, 70, 74). Regardless of the source, preQ_1_ is incorporated by the *tgt* gene product (tRNA-guanine transglycosylase) into the wobble position of specific tRNAs, where it is modified into Q by *queA* and *queG* gene products (136). *C*, covariation models of the three class I preQ_1_ riboswitch subtypes based on (69) and rfam.org/family/RF00522. Y symbolizes purine and R is pyrimidine. PreQ_1,_ prequeosine_1_.

Riboswitches are posited to be descendants of ancient regulatory systems (1) based on their presence in taxonomically diverse genomes, their utilization of multiple gene-regulatory strategies, and their ability to function without the assistance of proteins (14). More than 60 classes of extant riboswitches have been identified that bind to a variety of ligands including: amino acids, nucleic acid derivatives, monovalent or multivalent ions, guanidine, sugars, and other small-molecule effectors (2, 10, 15, 16, 17, 18). Analysis of riboswitch sequences supports a power-law relationship, suggesting that the total number of classes in the biosphere is at least two orders of magnitude greater than the number of classes currently identified (15). Many known riboswitches have spawned exquisite molecular structure-and-function studies, as well as innovative technologies. Biochemical techniques have quantified ligand-binding energetics and kinetics, revealing that most riboswitches are highly specific for their ligands (19, 20, 21, 22). Single-molecule fluorescence and force-probing have revealed the details of riboswitch dynamics, demonstrating that gene regulation can be controlled by kinetic as well as thermodynamic folding pathways (9, 23, 24, 25, 26, 27, 28). Functional assays have been developed to validate gene-regulatory activity *in vitro* (29, 30) and in live cells (24, 31, 32, 33, 34, 35, 36). Riboswitches have also inspired biotechnology applications, such as biosensors (37, 38, 39, 40) and ligand-programmable control elements for synthetic biology (41, 42).

Although many excellent reviews provide broad synopses of the field (2, 4, 15, 18, 43, 44, 45, 46), we focus here on the prequeosine_1_ (preQ_1_) (7-aminomethyl-7-deazaguanine) riboswitch family. The timeliness of this review is based on the confluence of several recent discoveries. Namely, the structure determination of the last remaining preQ_1_ riboswitch family member (47), the recent discovery of cooperative ligand binding by the most prominent member of the family (48), and the observation that preQ_1_ riboswitches can use noncanonical approaches to regulate transcription and translation (49, 50, 51). The preQ_1_ family comprises five members that adopt distinct three-dimensional folds that impart distinct modes of preQ_1_ recognition—including a novel mode of dual ligand binding. The family is also illustrative as a means to compare how different RNA folds sense the same ligand, which is an increasingly common theme in the field as new riboswitch classes are discovered (15). We will also examine a recurring structural motif found in many noncoding RNAs, known as the quintuple-base motif (QBM), which has been co-opted by riboswitches for ligand binding. We next examine how preQ_1_ binding leads to allosteric changes in a downstream expression platform to control gene expression at the levels of transcription and translation. Finally, we examine future challenges in the field, including a general need to identify riboswitches that do not use canonical expression-platform signals to control gene regulation. We also consider how riboswitch tertiary structure and resilience to mutations can influence the choice of a riboswitch as an antibacterial target. Promising antimicrobial leads have been found that target flavin mononucleotide (FMN) riboswitches (52, 53, 54) but the efficacy of such antibiotics was diminished by the rapid emergence of drug-resistance mutations. Importantly, any antibacterials that target a riboswitch with high specificity will experience the same issue. Finally, to expand the playing field of antibacterial targets, we consider the need to identify new targets starting from an evaluation of how a riboswitch influences bacterial fitness in a pathogenic context (55).

## A riboswitch family that senses preQ_1_

PreQ_1_ (7-deaza-7-aminomethylguanine) is the last soluble intermediate in the biosynthetic pathway for queuosine (Q)—a hypermodified nucleotide produced posttranscriptionally at position 34 of tRNAs containing GUN anticodons (Fig. 1*B*) (56). Q facilitates wobble pairing with U instead of C and confers translational fidelity (57) by allowing efficient incorporation of asparagine, aspartate, histidine, and tyrosine amino acids into the growing polypeptide chain (56). In bacteria, Q deficiency causes slow growth in mid-log phase (32), diminished viability under stressful growth conditions (58), increased resistance to nickel and cobalt but higher sensitivity to cadmium (59), and decreased virulence (60). Many eukaryotes require Q but cannot synthesize it *de novo*, necessitating its acquisition as a micronutrient from dietary sources or gut flora (61, 62, 63, 64). In germ-free mice, the absence of Q compromises tyrosine production (65). Many human cancers are deficient in Q-modified tRNAs (66, 67, 68), which portends greater malignancy (66, 68). The absence of *de novo* Q biosynthesis pathways in eukaryotes and the prevalence of preQ_1_ riboswitches in human pathogens (10, 69, 70) has elicited interest in targeting preQ_1_ riboswitches with drug-like molecules (71, 72).

A handful of metabolites are sensed by three or more riboswitch classes, such as guanidine (4 classes), SAM (7 classes), 2′-deoxyguanine (3 classes), and preQ_1_ (3 classes) (15). Although Q is not essential for viability in cell culture (32, 58), it is sufficiently important for cell survival in stationary phase (58) that multiple classes of riboswitches have evolved to sense the preQ_1_ metabolite. Like most riboswitches, those that sense preQ_1_ were found in bioinformatic searches designed to identify conserved secondary structures within noncoding sequences upstream of genes dedicated to preQ_1_ biosynthesis or transport (69, 70, 73, 74). These efforts led to the discovery of the distinct preQ_1_ riboswitch classes (I, II, and III). Due to their stable structures and high affinity for ligand, each class of the preQ_1_ riboswitch family has been studied both structurally and functionally (47, 48, 74, 75, 76, 77, 78, 79, 80, 81, 82, 83). Collectively, this work provides an opportunity to compare and contrast various RNA folds, modes of ligand binding, and mechanisms of gene regulation within a single riboswitch family.

## Class I riboswitches comprise three subgroups that bind one or two ligands

**Figure 2:**
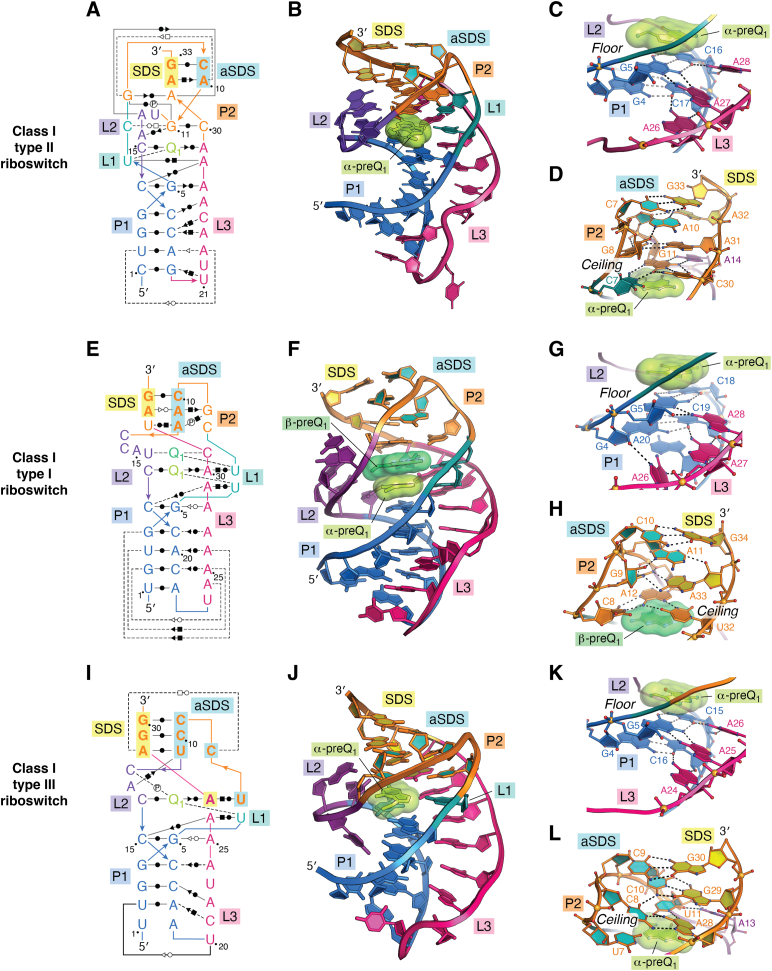
**Secondary structures, global folds, and expression platforms of class I preQ**_**1**_**riboswitches.***A*, secondary structure of the H-type pseudoknot (PK) from *Thermoanaerobacter tengcongensis* (*Tte*) based on cocrystal structures of preQ_1_-I_II_ riboswitches (79, 88). Here and elsewhere, colors correspond to specific PK pairing (P) or loop (L) sequences as defined (85); preQ_1_ is labeled “Q_1;_” base interactions are indicated by Leontis–Westhof symbols (139). The Shine-Dalgarno sequence (SDS) (expression platform) is highlighted *yellow*; the anti-(a)SDS is highlighted *cyan*. *B*, ribbon diagram of the *Tte* structure (PDB entry 3q50) (78). Helix P2 contains the aSDS-SDS pair (base and ribose rings filled *yellow and cyan*) that supports the gene-off conformation; the α-preQ_1_ ligand is depicted as a surface model (*green*). *C*, close-up view of the *Tte* riboswitch pocket floor showing the conserved G5-C16 pair in helix P1, whose sugar edge interacts with multiple adenines *via* A-amino kissing interactions (80). The view is rotated −180° about the *y*-axis from *panel B*. *D*, close-up view of the *Tte* riboswitch pocket ceiling showing canonical and noncanonical pairs in the aSDS–SDS interaction of P2. A C7^+^•G11-C30 triple forms directly above the α-preQ_1_ site. The view is rotated 90° about the *y*-axis from *panel C*. *E*–*H*, diagrams comparable to *A*–*D* for the *Carnobacterium antarticum* (*Can*) preQ_1_-I_I_ riboswitch (PDB entry 8fb3) (49). *I*–*L*, Diagrams comparable to *panels A*–*D* for the *Escherichia coli* (*Eco*) preQ_1_-I_III_ riboswitch (PDB entry 8fza) (47).

Class I riboswitches were identified originally in bioinformatic searches as small structured motifs (73) linked to Q biosynthesis genes (74, 84). Although all class I riboswitches were predicted to fold as simple H-type pseudoknots (reviewed in (85)), sequence differences initially led to the classification of two subgroups (types I and II). Type I riboswitches (abbreviated I_I_ for class I type I) are the most abundant preQ_1_ riboswitch group based on 1577 reported sequences (Fig. 1*C*, *left panel*) derived from a broad number of phyla including, Fusobacteriota, Actinomycetota, Chlorobiota, Bacteroidota, Bacillota, and Pseudomonadota (69, 74). In preQ_1_-I_I_ riboswitches, the 3′-strand of helix P1 (*i.e.*, pairing region 1) is always preceded by UC (Fig. 1*C*, *left panel*, chevron)—which is a key aspect of preQ_1_ recognition (see below). Type II riboswitches (abbreviated I_II_ for class I type II) have a similar phylogenetic distribution but show fewer sequences in the Bacillota and Pseudomonadota phyla. PreQ_1_-I_II_ riboswitches show a distinct consensus model derived from 521 sequences wherein the 3′-strand of helix P1 is preceded by AC (Fig. 1*C*, *middle panel*, chevron). Mutation of the latter cytosine in the *Bacillus subtilis* (*Bsu*) aptamer suggested that this base controls ligand specificity by Watson–Crick (WC) base pairing to the guanine-like face of preQ_1_ (74). The *Bsu* preQ_1_-I_II_ riboswitch regulates transcription (*e.g.*, Fig. 1*A*), and its aptamer structure was determined by NMR and crystallography (80, 83). (Details about transcriptional regulation are provided below). Our lab independently solved the crystal structures of the translation-regulating *Thermoanaerobacter tengcongensis* (*Tte*) riboswitch in the ligand-bound states with preQ_0_ and preQ_1_, as well as the ligand-free (apo) state. The *Tte* structures revealed two Shine-Dalgarno sequence (SDS) nucleotides buried inside a compact core wherein >50% of all bases in the riboswitch engage in noncanonical triple or quadruple base-pairing interactions (Fig. 2, *A* and *B*) (78, 79).

Structures of the preQ_1_-I_II_ *Bsu* and *Tte* aptamers were the first members of the preQ_1_ riboswitch family to be determined and are among the smallest known riboswitch folds. Each structure revealed a single ligand bound between two coaxially stacked helices wherein helix P1 forms a canonical A-form helix followed by an A-rich loop (L3); adenines in the latter loop use their N6-amino groups to donate hydrogen bonds to the P1 minor-groove, dubbed “A-amino kissing” interactions (80) (*e.g.*, Fig. 2, *A* and *C*). Although helix P2 of the *Bsu* aptamer forms through canonical WC pairing, only two of four P2 steps form canonical pairs in the *Tte* aptamer (Fig. 2, *A* and *D*). These structures provided a firm foundation for additional studies on the folding (80, 86, 87), switching activity (86, 87, 88), effector specificity (74, 78), and targeting of preQ_1_-I riboswitches with drug-like molecules (71, 72).

Although significant advances in our understanding of riboswitches came from investigations of the preQ_1_-I_II_ subgroup, no work was conducted on the type I or type III subgroups for over a decade, despite their different consensus models (Fig. 1*C*, *left* and *right panels*). This oversight was likely caused by the misclassification of the *Tte* riboswitch as a type I sequence (74), even though it clearly obeys the type II consensus model; that is, the 3′-strand of helix P1 is preceded by AC (Fig. 1*C*, *middle panel*, chevron and Fig. 2*A*). By contrast, a conserved UC motif occurs at the equivalent location in preQ_1_-I_I_ riboswitches (Fig. 1*C*, *left panel*, chevron), as well as an opposing UU motif atop the 5′ strand of P1, and a conserved C in the 3′-tail (Fig. 1*C*, *left panel*, asterisk and triangle). While investigating these sequence differences, our lab made the surprising discovery that preQ_1_-I_I_ riboswitches sense two preQ_1_ equivalents based on isothermal titration calorimetry (ITC) (48). This unexpected finding was supported by a cocrystal structure of the riboswitch from *Carnobacterium antarticum* (*Can*) wherein two head-to-tail preQ_1_ molecules stack to complete the coaxial helical fold that sequesters the first two SDS bases (Fig. 2, *E* and *F*). The binding pocket floor of preQ_1_-I_I_ riboswitches comprises a P1 helix stabilized by flanking A-amino kissing interactions, analogous to preQ_1_-I_II_ riboswitches (Fig. 2*G*). Similarly, preQ_1_-I_I_ riboswitches also bury only the first two SDS nucleotides in helix P2, reminiscent of the *Tte* preQ_1_-I_II_ riboswitch. However, the type I ceiling must allow two stacked ligands. Both metabolites are accommodated by noncanonical interactions at the base of helix P2. Specifically, the ceiling directly above the β-site is a C8•A12•U32 base triple that contains a *trans* A•C pair (Fig. 2*H*) that widens the backbone compared to preQ_1_-I_II_ and preQ_1_-I_III_ riboswitches. Although several riboswitches were found previously to recognize multiple ligands, their modes of binding utilized two separate pockets without ligand-to-ligand contact (89, 90, 91, 92, 93, 94, 95, 96). Hence, the direct stacking of two preQ_1_ metabolites in one pocket is unprecedented not only in the preQ_1_ riboswitch family, but among all known riboswitches. Moreover, both preQ_1_ molecules were shown to be necessary for gene regulation (48), whereas other dual-ligand riboswitches appear to rely primarily on binding at one site for gene regulatory function (92, 97, 98). A more detailed description and comparison of binding pockets is provided below.

The preQ_1_-I_III_ riboswitches were identified several years after the initial discovery of preQ_1_-I_I_ and preQ_1_-I_II_ riboswitches. The type III subgroup originated from an updated analysis of class I covariation models using 293 sequences found almost exclusively in the Pseudomonadota phylum (69). This subgroup has the smallest, most stringent covariation model, and contains a pyrimidine-rich stem–loop (Fig. 1*C*, *right panel*) (69). Unlike other subgroups, the type III consensus model convincingly suggests occlusion of an AAGG sequence (*i.e.*, the SDS expression platform) *via* WC pairing (P) in the H-type pseudoknot (Fig. 1*C*, *right panel*, triangle). In addition, preQ_1_-I_III_ riboswitches exhibit a strongly conserved UU motif flanking the 5′-strand of P1—reminiscent of type I—but an AC motif at the specificity base (Fig. 1*C*, *right panel*, asterisk and chevron), akin to type II. To test this subgroup for ligand binding, our lab analyzed various sequences by ITC, which definitively revealed a 1:1 ligand-to-receptor ratio (20, 47). Based on the covariation model, a type III sequence was identified from *Escherichia coli* (*Eco*), which yielded a cocrystal structure. The resulting atomic model confirmed the pseudoknot fold, the 1:1 preQ_1_:RNA stoichiometry, a pocket floor that resembles preQ_1_-I_I_ and preQ_1_-I_II_ riboswitches, and complete burial of the SDS (Fig. 2, *I*–*L*) (47). In contrast to preQ_1_-I_I_ riboswitches, type II and type III ceilings arise from base quadruples centered around canonical G-C and A-U pairs (Fig. 2, *D*, *H* and *L*). In both the *Tte* preQ_1_-I_II_ and *Eco* preQ_1_-I_III_ aptamers, the major-groove edge of the canonical pair in the ceiling forms a *trans* interaction with a cytosine, whereas the sugar-edge of the canonical pair interacts with the WC face of an L3 adenine. Interestingly, the *Bsu* preQ_1_-I_II_ aptamer ceiling is a simple C-G WC pair (83), which may be better for promoting formation of the downstream terminator hairpin when preQ_1_ levels are high (74), as compared to SDS burial that must withstand ribosomal helicase activity [reviewed in (99)]. (Both concepts are discussed below).

## The quintuple base motif: a recurring pattern in H-type pseudoknots with base triples

**Figure 3:**
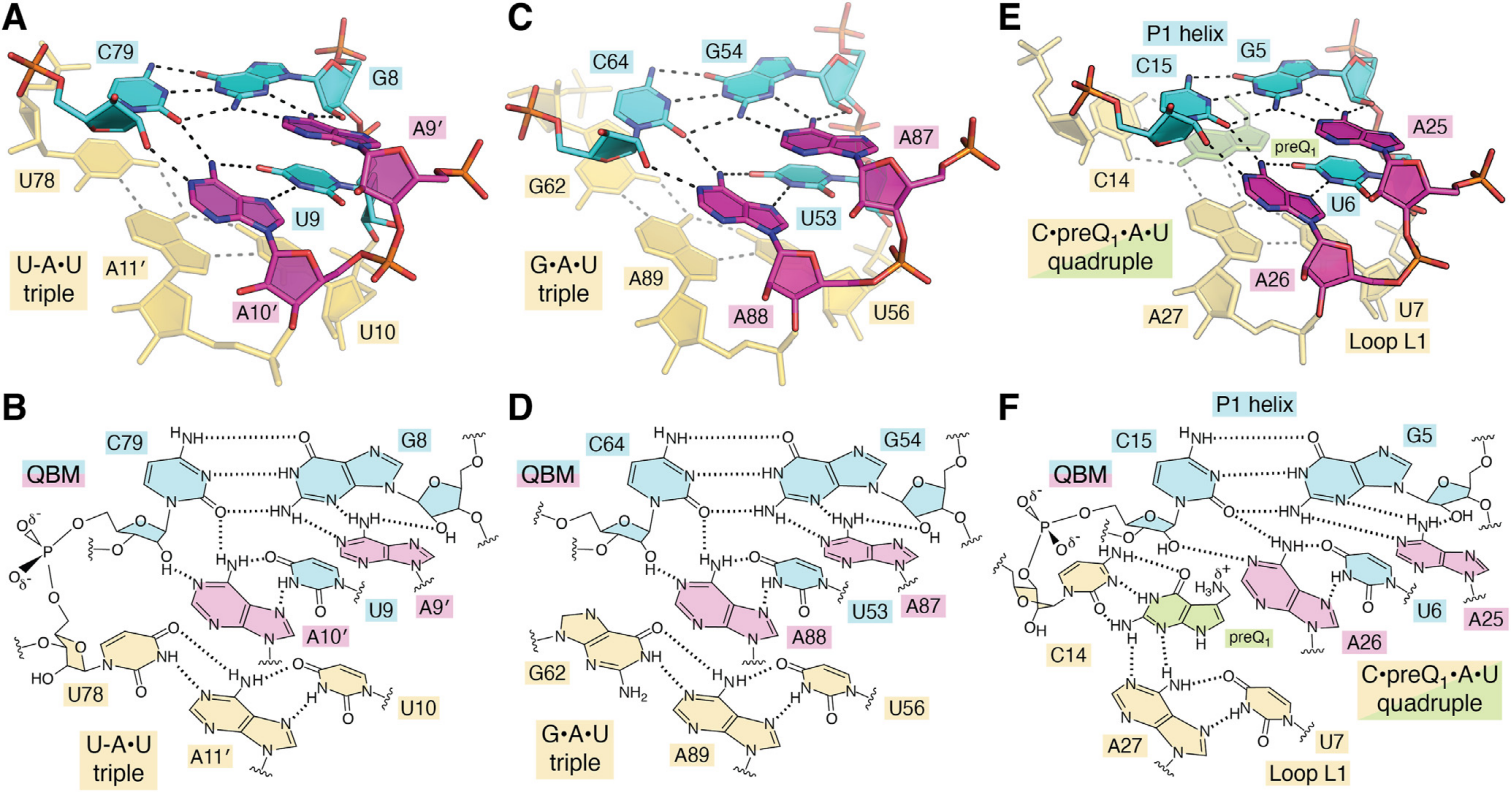
**The recurring quintuple-base motif transitions A-rich sequences interacting with the minor groove to major-groove base triples.***A*, a QBM from the double-ENE RNA stability fold in complex with a 28-mer poly(A) RNA (PDB entry 7jnh) (100). An upper A-form helix (*cyan*) uses minor-groove interactions with adenines (*purple*) to transition to a new helix (*pale yellow*) stabilized by a major-groove U-A•U triple. Nucleotides of the QBM are colored *cyan* and *purple*. *B*, schematic diagram of QBM interactions in *panel A*. *C*, QBM of *Campylobacter jejuni* Cas9 in complex with single-guide RNA (101) (PDB entry 5x2g). Colors are similar to *panel A*. *D*, schematic diagram of QBM interactions in *panel C*. *E*, QBM of the *Eco* preQ_1_-I_III_ riboswitch (PDB entry 8fza). Transition to the underlying base triple (*pale yellow*) has been altered to bind preQ_1_ through a base quadruple, located beneath the QBM; this ligand-recognition feature is observed in all class I preQ_1_ riboswitches. *F*, schematic diagram of QBM interactions in *panel E*. ENE, element for nuclear expression.

Despite their small size, class I riboswitches comprise multiple recurring structural motifs that compose the overall pseudoknot fold (85). One previously overlooked element is the QBM. This structural module transitions an H-type pseudoknot from A-rich sequences in the helical minor-groove to a series of underlying major-groove base triples. Although present in the original preQ_1_-I_II_ riboswitch structures from *Bsu* and *Tte* (79, 81, 83), the QBM was identified only recently as a recurring motif when it was observed in the rice transposase TWIFB1 dENE-poly(A)_28_ complex (100) (Fig. 3, *A* and *B*). A broad structural survey revealed the QBM is present in other folds such as the Cas9 *trans*-activating crRNA complex (101) (Fig. 3, *C* and *D*), as well as other pseudoknotted RNA molecules including riboswitches. We observed that all bound-state class I riboswitches utilize a QBM, which forms a transition point between helix P1 and the binding pocket (47, 48, 79, 80, 83, 88). The *Eco* preQ_1_-I_III_ riboswitch exemplifies a QBM co-opted for ligand recognition. Specifically, a G-C canonical pair in A-form helix P1 hydrogen bonds through its minor-groove to conserved adenines, A25 and A26, in the A-rich (L3) loop (Fig. 3, *E* and *F*). A third L3 adenine, A27, interacts through its major-groove edge to conserved U7 in loop L1. The latter interaction supports the QBM motif but A27 simultaneously hydrogen bonds to preQ_1_ in a manner that replaces the expected cross-strand base pair, equivalent to U78-A11′ in the dENE-poly(A)_28_ complex and G62•A89 in the Cas9–crRNA complex (Fig. 3, *A*, *C* and *E*). Hence, preQ_1_ supports the QBM by altering hydrogen bonding in the lower base triple to form a C•preQ_1_•A•U quadruple that is integral to ligand recognition and QBM stability (Fig. 3*F*, *yellow and green layer*). Interestingly, the SAH riboswitch also uses a QBM (100, 102), although the motif did not evolve to integrate the adenine base of the ligand into a quadruple (not shown) as observed for preQ_1_-I riboswitches (Fig. 3*F*). Interestingly, the QBM is also used by the preQ_1_-I_I_ riboswitch to bind two preQ_1_ ligands. Although binding to the first ligand is analogous to the preQ_1_-I_III_ riboswitch (Fig. 3, *E* and *F*), the second preQ_1_ molecule interacts with the major-groove edge of U7 and requires an additional specificity base, C31, to read out the ligand’s minor-groove edge (not shown). The latter C31 nucleotide interrupts the stretch of adenines in the preQ_1_-I_I_ riboswitch 3′-tail (Fig. 1*C*, *left panel, triangle*), illustrating how the QBM can be co-opted to add a new ligand binding site with an altered mode of recognition. Due to topology differences between H-type pseudoknots and HL_out_ pseudoknots (reviewed in (85)), class II and III preQ_1_ riboswitches do not exhibit QBMs. Instead, preQ_1_ sits directly atop two or three tiers of U-A•U or U-A•A major-groove base triples. Transition from the triple helix to the binding pocket occurs instead *via* inclined A-minor bases (discussed below). QBM prevalence in H-type pseudoknots—including the preQ_1_-I riboswitches highlighted here—supports its inclusion among other recurring RNA folding motifs such as T-loops (103), A-minor patches (104), and k-turns (105).

## Class II preQ_1_ riboswitches integrate the SDS into the floor of the aptamer pocket

**Figure 4:**
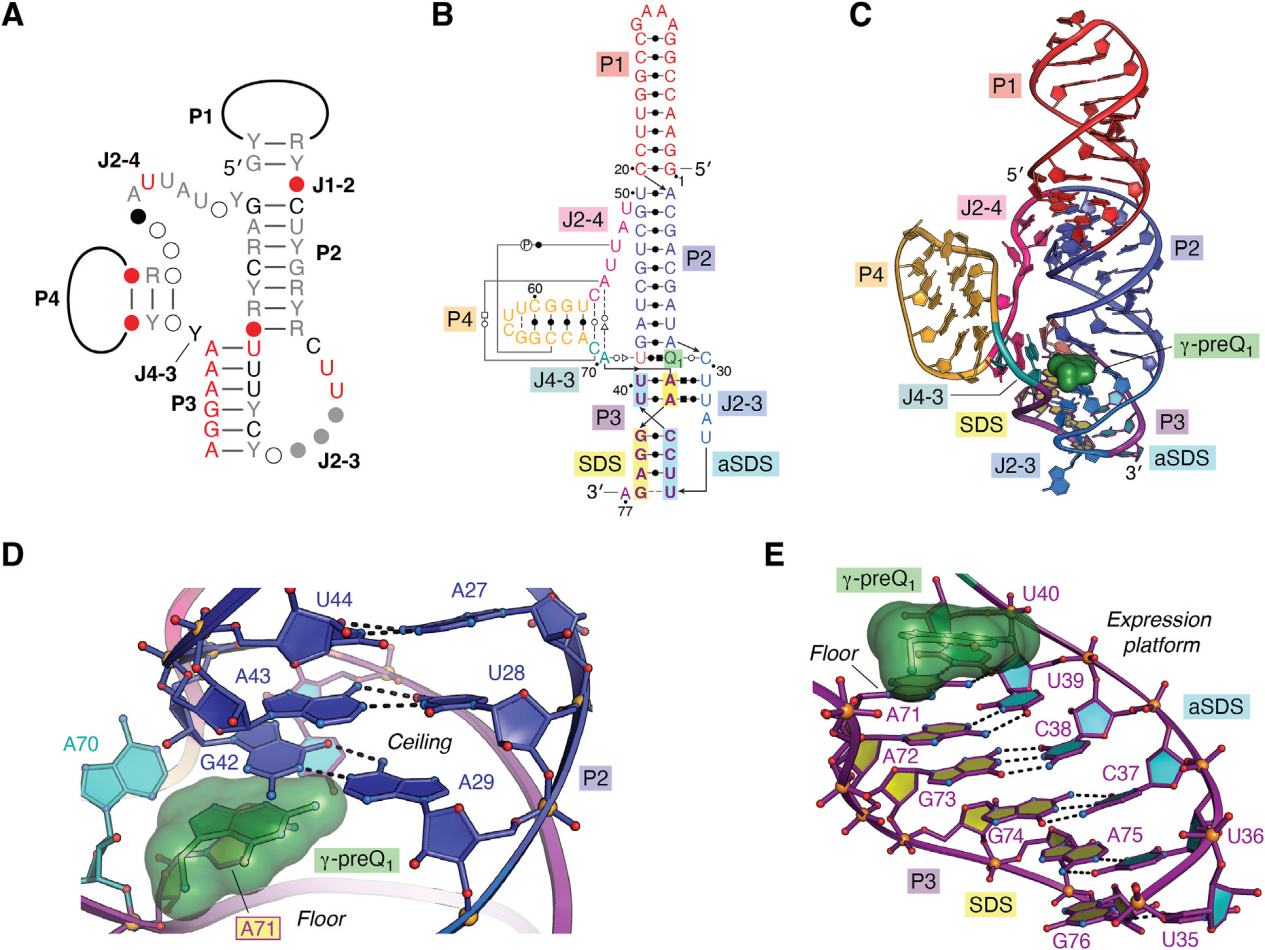

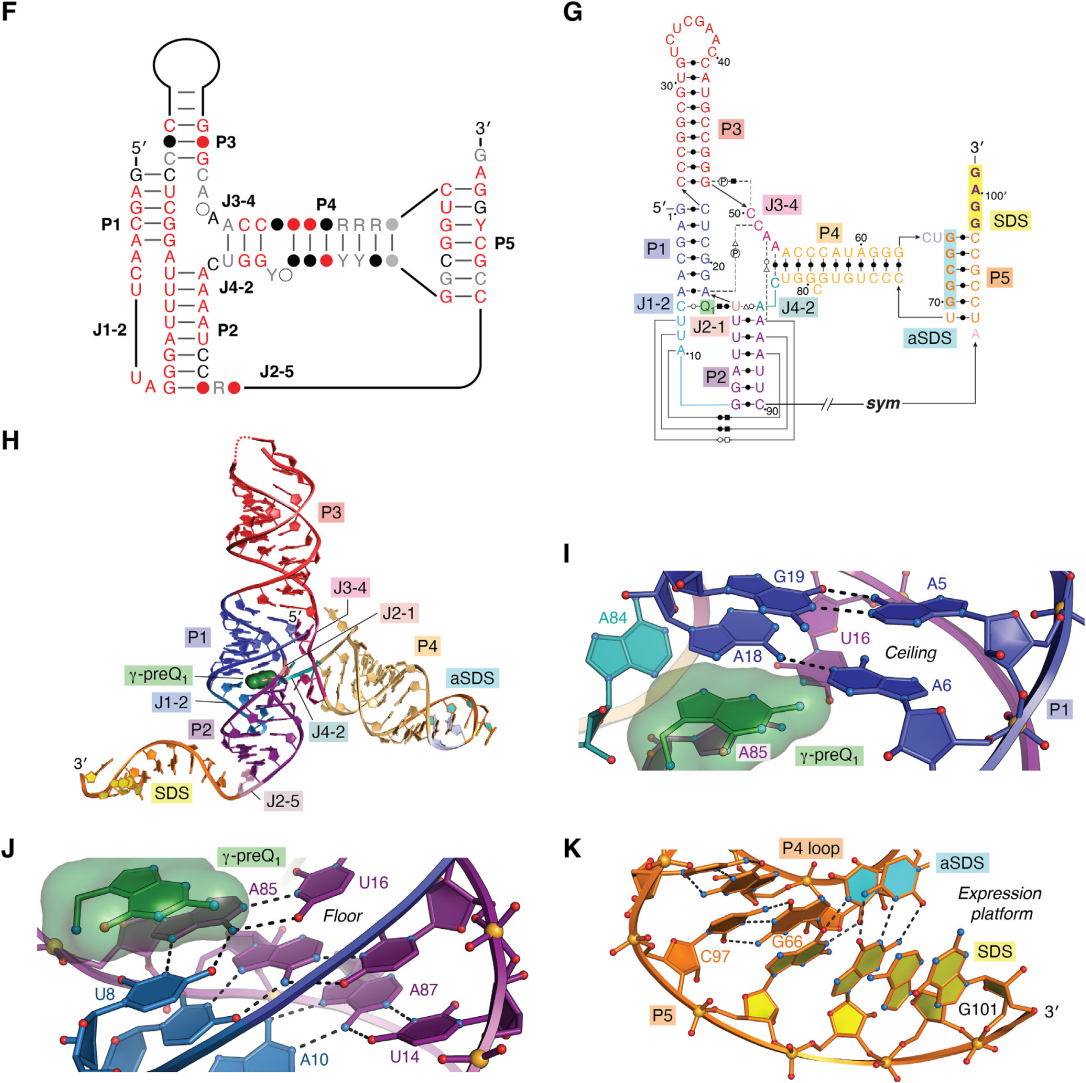
**Covariation models, secondary structures, global folds, and expression platforms of class II and class III preQ**_**1**_**riboswitches.***A*, covariation model of the preQ_1_-II riboswitch based on (69, 70) and rfam.org/family/RF01054. *B*, secondary structure based on the *Lactobacillus rhamnosus* (*Lrh*) cocrystal structure (PDB entry 4jf2) (76). *C*, ribbon diagram depicting the HL_out_ PK tertiary fold of the *Lrh* riboswitch. The riboswitch displays a novel mode of preQ_1_ recognition compared to class I, dubbed the “γ” mode if binding. *D*, the ceiling of the preQ_1_ binding pocket. *E*, the expression platform comprises the SDS paired with the aSDS, forming helix P3; the first base of the SDS, A71, forms the floor of the binding pocket directly below preQ_1_ (*semitransparent green surface*) (76). (**cont’don next page**) *F*, covariation diagram of preQ_1_-III riboswitches based on (69) and rfam.org/family/RF02680. *G*, secondary structure based on the *Faecalibacterium prausnitzii* (*Fpr*) cocrystal structure (PDB entry 4rzd). The paired aSDS-SDS helix P5 is based on the observed crystal contact; the predicted aSDS (*cyan*) and SDS (*yellow*) are based on the consensus model in *panel F*. *H*, ribbon diagram of the HL_out_ PK tertiary fold of the *Fpr* riboswitch (77). The γ-mode of preQ_1_ binding is similar to the preQ_1_-II riboswitch. *I*, the ceiling of the preQ_1_ binding pocket is made by an A18•A6 pair in helix P1. *J*, The floor of the binding pocket comprises three underlying layers of major-groove base triples at the interface between J1-2 and P2. *K*, Computational model of the P5 helix expression platform based on targeted molecular dynamics starting from the co-crystal structure in panel *H* (77). The first two bases of the SDS are buried. PK, pseudoknot; SDS, Shine-Dalgarno sequence; preQ_1_, prequeosine_1_.

The preQ_1_-II (class II) riboswitch was identified by a bioinformatic search that showed a distinct covariation model in the 5′-UTR of genes predicted to encode a membrane protein classified as COG4708 or DUF988 (now referred to as *queT*) (106). The class II motif identified in 72 sequences was confirmed experimentally to recognize preQ_1_ (70). Class II preQ_1_ riboswitches are limited to the Bacillota phylum and comprise sequences primarily from the *Streptococcaceae* and *Lactococcus* families (69, 70). This phylogenetic distribution is much narrower than preQ_1_-I riboswitches. The consensus model reveals that the class II aptamer is nearly twice the size of preQ_1_-I riboswitches and folds as an HL_out_ pseudoknot comprising four conserved pairing regions (P1–P4), and four intervening junctions (J) (Fig. 4*A*) (106). Formation of P3 was predicted to occlude the SDS, leading exclusively to control of translation. Following its discovery, a cocrystal structure of a representative preQ_1_-II riboswitch from *Lactobacillus rhamnosus* (*Lrh*) was solved by our lab (76). Subsequently, a class II *Streptococcus pneumoniae* (*Spn*) riboswitch structure was determined independently using NMR, which confirmed the HL_out_ pseudoknot fold, the mode of preQ_1_ binding, and complete occlusion of the SDS (81).

Both structures revealed preQ_1_ nestled between helices P2 and P3 where the ligand completes a coaxial stack comprising P1, P2, and P3 (Fig. 4, *B* and *C*), analogous to ligand-mediated stacking in preQ_1_-I riboswitches (Fig. 2). In contrast to preQ_1_-I riboswitches, the class II binding pocket ceiling comprises a nonconserved G-A pair (Fig. 4*D*) that is replaced by a naturally occurring WC G-C base pair in the *Spn* riboswitch (70, 81). Helix P4 is oriented orthogonally to the P1-P2-P3 helical stack and does not play a direct role in preQ_1_ readout. Deletion of P4 showed only a minor effect on preQ_1_ binding (75, 107). However, single-molecule FRET (smFRET) studies revealed that P4 plays a critical role in the kinetics of preQ_1_ binding and the ability to bind preQ_1_ on a short time scale (107). This work as well as independent NMR analysis (81) demonstrated that sequences adjacent to the binding pocket play a key role in riboswitch dynamics and pseudoknot stability.

Because preQ_1_-II riboswitches were predicted to regulate translation, it is significant that the *Lrh* cocrystal structure shows the complete 6-nt SDS sequestered *via* canonical WC pairing within helix P3, located directly beneath the preQ_1_ binding pocket (Fig. 4, *B*, *C* and *E*) (76). This observation strongly links preQ_1_ sensing to P3 expression platform stabilization. Complementary smFRET experiments (107, 108), in-line-probing (70, 76), and chemical modification assays (32) corroborated the observed mode of ligand-dependent SDS occlusion by demonstrating that P3 becomes more flexible in the absence of preQ_1_.

## Class III riboswitches position the expression platform far from the preQ_1_ pocket

PreQ_1_-III riboswitches are the most recently discovered class in the preQ_1_ riboswitch family and are the least characterized family member in terms of their mechanism of action. Class III riboswitches were identified in a bioinformatic search of 5′-UTRs associated with the preQ_1_ transporter gene *queT* (69). A novel consensus model was assembled from 86 sequence representatives that were mostly metagenomic (Fig. 4*F*). The remaining sequences were derived from the *Ruminococcaceae* family—among the most abundant bacteria in the intestinal microbiome of healthy adults (109).

At 100-nt in length, the class III riboswitch is the most complex preQ_1_-riboswitch described to date. PreQ_1_-III riboswitches comprise five WC-pairing regions (P), and four well-defined junction regions (J) (Fig. 4*F*). The structure was predicted to form two multibranch pseudoknots, distinguishing it from simpler H-type and HL_out_ pseudoknots of preQ_1_-I and preQ_1_-II riboswitch classes (69). The first pseudoknot region at P1 forms between the 5′-sequence and the junction between P2 and P3. The second pseudoknot forms helix P5 using nucleotides in the 3′-tail that contain the SDS, and those of the P4 stem–loop, which contribute the anti-(a)SDS. Subsequently, a cocrystal structure of a representative class III riboswitch from *Faecalibacterium prausnitzii* (*Fpr*) was solved by our lab revealing the 5′-pseudoknot can be classified as an HL_out_ variety wherein P3 is considered an extension of the J3-4 loop (Fig. 4, *G* and *H*) (77). As observed for other preQ_1_ riboswitch family members, the ligand completes coaxial helical stacking, since P1 lies between helices P2 and P3. The pocket ceiling comprises a highly conserved *trans* A•A base pair that stacks on preQ_1_ (Fig. 4, *F* and *I*), and is distinct compared to ceilings in the class I and II preQ_1_ riboswitches. Noncanonical pairing in the class II and class III ceilings contributes flexibility to the pocket based on molecular dynamics and chemical modification analysis (76, 110, 111), which likely influences preQ_1_ association and dissociation rates. The pocket floor of the preQ_1_-III riboswitch comprises multiple layers of U-A•U/A major-groove base triples (Fig. 4, *G* and *J*), reminiscent of the class II preQ_1_ riboswitch (Fig. 4*B*). Such base triples stabilize the fold while providing a flat surface that stacks with the heterocyclic rings of preQ_1_.

Although the consensus model predicts a second pseudoknot in the expression platform that partially buries the SDS through formation of the P5 helix (Fig. 4*F*), the crystal structure did not reveal this expected helical pairing mode (Fig. 4*H*) (77). Instead, the P4 loop and 3′-tail engaged in an intermolecular crystal contact (Fig. 4*G*), most likely caused by the high concentration of RNA used for crystallization and the dynamic nature of the expression platform. Accordingly, the intermolecular P5 helix observed in the crystal structure does not represent the biological conformation. In-line probing experiments and *in vitro* selective 2′-hydroxyl acylation analyzed by primer extension (SHAPE) analysis of the *Fpr* preQ_1_-III riboswitch independently showed strong preQ_1_-dependent modulation of P1 and P2, indicating that the ligand influences the conformation of the pseudoknotted pocket (69, 77). However, P5 modulation was not observed, suggesting that formation of the aSDS-SDS helix was too fast to be captured by chemical modification approaches. To explore the feasibility of expression platform occlusion through P5 helix formation, a computational model was prepared starting from the *Fpr* class III crystal structure (Fig. 4*K*). Modeling supports the predicted mode of aSDS-SDS pairing in the consensus model (Fig. 4*F*), which required a scissor-like motion in which helix P4 pivots toward the P2-P1-P3 coaxial stack (77). This results in a second pseudoknot—which can be considered a partially nested H-type pseudoknot within the HL_out_ pseudoknot—that composes the gene-regulatory aSDS-SDS helix P5 (Fig. 4, *F* and *K*). The ability of the aSDS to dock with the 3′-tail is supported by smFRET experiments, which revealed that saturating levels of preQ_1_ increase the population of riboswitch molecules that rapidly sample the docked and undocked state of P5 (77). Collectively, these findings support the hypothesis that the *Fpr* riboswitch controls translation by reducing ribosomal access to the SDS upon ligand binding (described below). A novel aspect of the preQ_1_-III riboswitch is the long distance between the preQ_1_ pocket and the expression platform. This arrangement contrasts sharply with class I and II riboswitches, which integrate SDS pairing into the aptamer. A second unique aspect of the preQ_1_-III riboswitch is that aSDS-SDS docking is stimulated by preQ_1_ binding in a process that is highly dynamic. In fact, SDS undocking is 2-fold faster than docking. Accordingly, class III riboswitches appear to be controlled by dynamic conformational switching, rather than equilibrium folding and occlusion of the SDS as observed for class II riboswitches (Fig. 4*E*).

## Three different preQ_1_ recognition modes and strategies to reduce off-target binding

**Figure 5:**
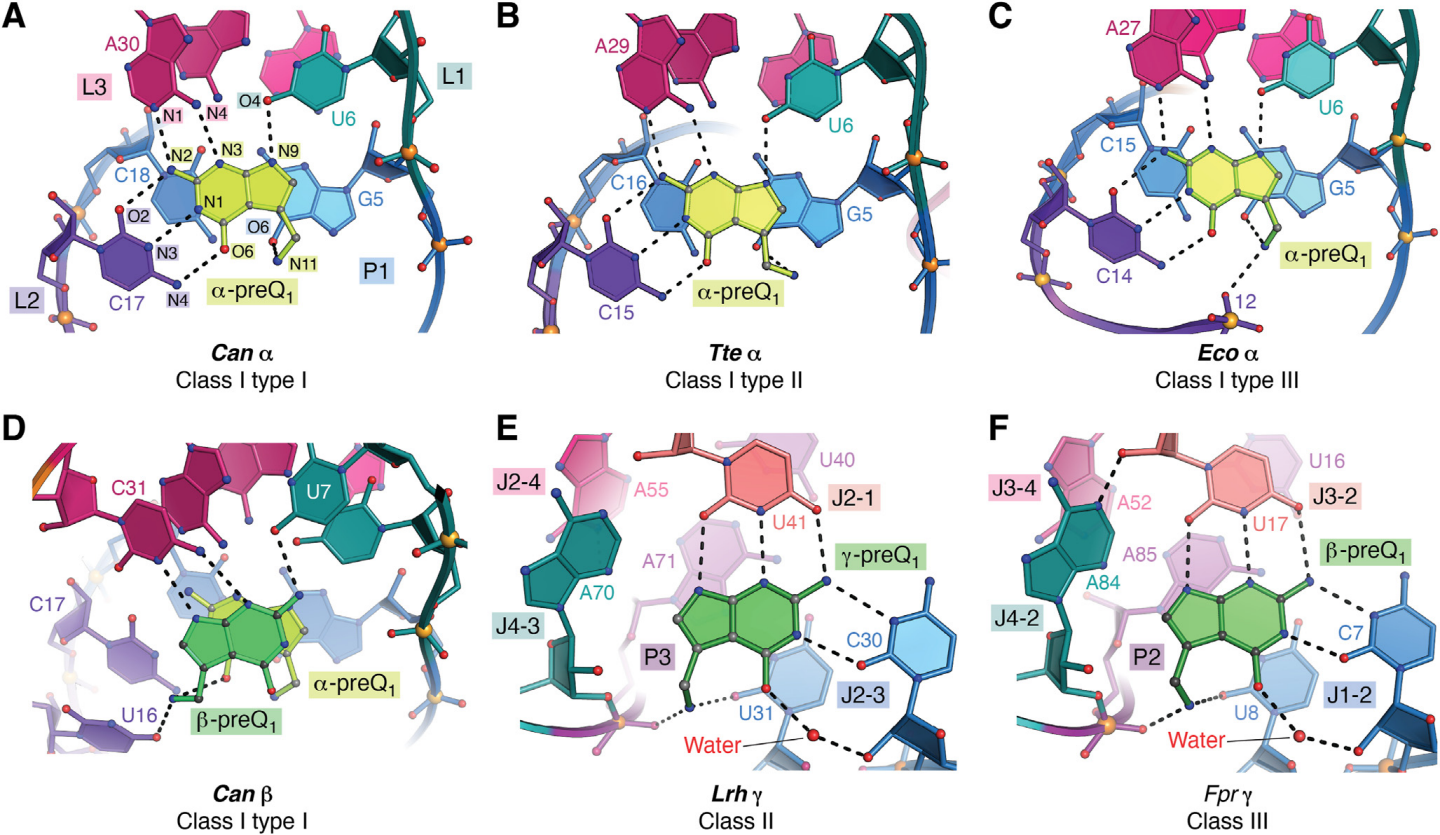
**Overview of the α, β, and γ modes of ligand binding by the three classes of preQ**_**1**_**riboswitches.***A*–*C*, the most common mode of preQ_1_ recognition, α, occurs in all class I preQ_1_ riboswitches as exemplified by the *Can* preQ_1_-I_I_, *Tte* preQ_1_-I_II_, and *Eco* preQ_1_-I_III_ riboswitch binding pockets. The guanine-like face of preQ_1_ is recognized by a strictly conserved cytosine specificity base that uses WC pairing (*e.g.*, C17 in the *Can* preQ_1_-I_I_ riboswitch). Each pocket contains a conserved WC-base–paired floor, equivalent to the *Can* riboswitch G5-C18 pair. Conserved bases recognize the minor-groove-edge equivalent of preQ_1_. In all types, the preQ_1_ methylamine group hydrogen bonds with the O6 keto moiety of guanine in the pocket floor. The *Eco* preQ_1_-I_III_ riboswitch also shows hydrogen bonding to the nonbridging phosphate of position 12. *D*, the β-mode of recognition has been observed only in preQ_1_-I_I_ riboswitches, as exemplified by the *Can* riboswitch. Unlike the α-mode of recognition, there is no specificity base at the WC face of the β ligand. The minor-groove-edge equivalent of preQ_1_ is recognized by conserved bases. The methylamine group of preQ_1_ hydrogen bonds to the O4 keto of U16 and the O6 keto group of the underlying α-preQ_1_, which stacks beneath the β-preQ_1_ ligand. *E* and *F*, the γ-mode of recognition represents a third unique way to bind preQ_1_. This recognition pattern is characterized by a conserved *trans*–WC interaction between a cytosine base of the RNA pocket and the guanine-like face of preQ_1_, as exemplified by preQ_1_-II and preQ_1_-III riboswitches. A water molecule at the O6 keto oxygen of preQ_1_ mediates a contact with the 2′-hydroxyl of the specificity base. A conserved uracil base from the pocket reads the minor-groove edge equivalent of preQ_1_. Watson–Crick; preQ_1_, prequeosine_1_.

The selectivity needed to form a ligand-binding pocket usually allows only minute deviations during evolution, resulting in high sequence conservation of the aptamer domain (112). When mutations do occur at highly conserved positions in aptamer pockets, the resulting variant may have evolved a different ligand-binding preference (113, 114, 115, 116). Examples include “snuggler” riboswitches (15, 117), such as those found among guanine-I riboswitch sequences wherein mutations exist at key ligand-recognition bases. The resulting riboswitches bind to an alternative ligand, 8-oxoguanine (11), which arises through oxidative DNA damage. By contrast, each type of class I riboswitch binds the same ligand, even though the aptamer consensus models differ sufficiently to warrant classification as three distinct subgroups (Fig. 1*C*) (69). Despite this, each class I aptamer shows similar ligand recognition features that confer high affinity and specificity for preQ_1_. Namely, each type of class I riboswitch uses a cytosine specificity base—equivalent to C17 in the *Can* preQ_1_-I_I_ riboswitch—that hydrogen bonds to preQ_1_ in a *cis* WC-pairing manner (Fig. 5, *A*–*C*). Additional conserved interactions include U6 in loop L1, which recognizes the N9 imino of preQ_1_, and A30 in the A-rich L3 loop, which recognizes preQ_1_ through its N3 and N2 positions. The latter RNA bases confer a hydrogen bond acceptor-donor-acceptor pattern that is specific for the minor-groove-edge (equivalent) of preQ_1_. Analogous interactions are observed in preQ_1_-I_II_ and preQ_1_-I_III_ riboswitches.

Class I interactions with the preQ_1_ 7-aminomethyl group vary slightly. All types (I, II, and III) hydrogen bond to the O6 keto of a guanine in the pocket floor, which forms the closing pair of helix P1 (Fig. 5, *A*–*C*); the *Bsu* preQ_1_-I_II_ aptamer shows similar hydrogen bonding (80, 83). This interaction is consistent with G5 conservation in the consensus models (Fig. 1*C*), as well as mutagenesis data wherein replacement of the O6 keto group with an exocyclic amine severely impaired ligand binding by the *Tte* preQ_1_-I_II_ and *Eco* preQ_1_-I_III_ aptamers (47, 78). The *Eco* preQ_1_-I_III_ aptamer also makes an electrostatic interaction to the position 12 phosphate backbone (Fig. 5*C*), reminiscent of the *Bsu* preQ_1_-I_II_ aptamer (83). The latter interaction is favored by the *Eco* and *Bsu* aptamers because each forms a canonical P2 helix that bends the backbone sharply to interact with the preQ_1_ 7-aminomethyl group. Overall, the latter set of interactions compose the “α” binding mode for preQ_1_ (48), which is the principal ligand recognition pattern observed among all class I preQ_1_ riboswitches.

PreQ_1_-I_I_ riboswitches possess an additional binding site that uses a unique mode of ligand recognition known as “β.” The β site is adjacent to the α site and has not been observed as a standalone mode of ligand recognition. Binding at the β site does not use WC readout of preQ_1_, but instead employs a highly conserved C in the 3′-tail of the riboswitch (Fig. 1*C*, *left panel*, *triangle*) that reads the ligand’s minor-groove-edge equivalent (Fig. 5*D*) (noted above as C31 in the QBM discussion). A second conserved uracil (U7) in L1 hydrogen bonds to the preQ_1_ exocyclic amine. The α and β preQ_1_ sites stack on each other in a head-to-tail orientation, and the β-preQ_1_ 7-aminomethyl group hydrogen bonds to the α-preQ_1_ O6 keto group. Analysis of preQ_1_ binding in preQ_1_-I_I_ riboswitches indicates that they utilize positive cooperativity. Specifically, the binding of the first site enhances affinity for the second site and *vice versa*. Binding of preQ_1_ to the type I aptamer is described best by a model with two interdependent, nonequivalent sites (118). In this manner, it was possible to measure a cooperativity constant, γ, for multiple preQ_1_-I_I_ riboswitches including *Carnobacterium antarcticum* (γ = 7.7), *Haemophilus influenzae* (γ = 26.7), and *Neisseria gonorrhea* (γ = 32.9) (48). These results indicate that members of the type I subgroup show high levels of positive cooperativity.

Although class II and class III preQ_1_ riboswitches adopt distinct HL_out_ pseudoknot folds (Fig. 4, *C* and *H*) (77) and show no clear phylogenetic connection (69), they exhibit ten identical nucleotides in their ligand-binding pockets. The unique mode of preQ_1_ recognition by class II and III pockets has been dubbed “γ.” Each pocket has a conserved cytidine that reads the preQ_1_ WC face using a *trans* interaction (*e.g.*, C30 in class II and C7 in class III) (Fig. 5, *E* and *F*). A water molecule completes the hydrogen bond pattern by mediating a contact from the O6 keto group of preQ_1_ to the 2′-hydroxyl group of C30 or C7. The water-mediated contact to the ligand explains how the class II riboswitch binds to the ligand 2,6-diaminopurine (70). The preQ_1_ minor-groove-edge equivalent is read out by a conserved uracil (*i.e.*, U41 in class II and U17 in the class III, Fig. 5, *E* and *F*). Like the two bases used in α and β recognition modes of class I riboswitches, a single uracil in class II and class III riboswitches provides a hydrogen bond acceptor–donor–acceptor interaction. Another feature shared by class II and III riboswitches is a pair of inclined, A-minor bases that pack against the pyrrole ring of preQ_1_. The adenine closest to preQ_1_ makes van der Waals contacts. This base (A70 in loop J4-3 of class II, Fig. 5*E* and A84 in loop J4-2 of class III, Fig. 5*F*) does not form a true A-minor interaction (76, 77) because it contacts the uracil-preQ_1_ step, which is not a true base pair. The more distal adenine (A55 in loop J2-4 of class II and A52 in loop J3-4 of class III) forms a genuine A-minor contact with the edge of the pocket floor, which helps to compose major-groove base triple U40-A71•U31 (class II) and U16-A85•U8 (class III) (Fig. 5, *E* and *F*). In both class II and class III pockets, the 7-aminomethyl group of preQ_1_ coordinates the phosphate of an adenine in the pocket floor (A71 in class II and A85 in class III) (Fig. 5, *E* and *F*). This interaction is a salt bridge because the 7-aminomethyl group of preQ_1_ is charged at neutral pH. The 7-aminoemethyl group also hydrogen bonds to the keto oxygen of a uracil base (U31 in class II and U8 in class III, Fig. 5, *E* and *F*).

The remaining three identical nucleotides shared by the class II and class III pockets are an underlying layer of U-A•U major groove bases triples (Fig. 4, *B* and *G*). Collectively, the ten common nucleotides superimpose with an average RMSD of 1.1 Å (77). However, whereas A71 of the class II riboswitch represents the first nucleotide of the SDS (Fig. 4*D*), the first nucleotide of the class III SDS is > 40 Å away from the binding pocket (Fig. 4*H*). This difference underscores the distinctive expression platforms used by these two members of the preQ_1_ riboswitch family.

The ability of class I preQ_1_ riboswitches to discriminate against non-preQ_1_ molecules has been shown previously for a variety of pyrrolopyrimidine analogs tested for binding to the *Bsu* preQ_1_-I_II_ aptamer (74). The preQ_1_-I_II_ binding pocket binds in order of affinity preQ_1_ > preQ_0_ > guanine in accord with the observed crystal structure of the α binding pocket (*e.g.*, Fig. 5*B*). In agreement with the crystal structures, the *Tte* preQ_1_-I_II_ riboswitch exhibited only a 17-fold difference in equilibrium binding between preQ_1_ and preQ_0_ (78). A kinetic analysis of the association (*k*_on_) and dissociation (*k*_off_) rates indicated that preQ_1_ binds 12-fold more rapidly than preQ_0_ but both ligands dissociate with a nearly equal rate. The cocrystal structure of the preQ_0_-bound state reveals that the linear nitrile group—which replaces the 7-aminomethyl group—points into solvent where it fails to hydrogen bond with the conserved guanine in the pocket floor (79). A more extreme case of kinetic discrimination was observed for preQ_1_ and preQ_0_ recognition by the *Eco* preQ_1_-I_III_ riboswitch, which binds preQ_1_ 130-fold more tightly than preQ_0_ (47). Whereas the association rate for preQ_1_ is 6.8-fold faster than preQ_0_, the dissociation rate for preQ_1_ is 20-fold slower (47). A model of preQ_0_ bound to the *Eco* preQ_1_-I_III_ aptamer reveals that the nitrile group is incapable of interacting with the phosphate backbone at C12 or the O6 keto of guanine in the pocket floor (47). This effect is due to the inability of the nitrile to donate a hydrogen bond as well as its linear bond geometry relative to the 7-aminomethyl group.

The capacity of preQ_1_-I_II_ and preQ_1_-I_III_ riboswitches to discriminate between two closely related metabolites might be explained by differences in the gene products controlled by these riboswitches. PreQ_1_-I_II_ riboswitches govern the expression of *queT* genes encoding an energy-coupling factor transporter that exclusively transports preQ_1_ (119, 120). Alternatively, bacteria that contain type III sequences lack *queT* but instead use a preQ_1_/preQ_0_ dual transporter *yhhQ* (69, 119). Since conversion of preQ_0_ to preQ_1_ consumes a single NADPH molecule per each preQ_0_ modified (84), it may be advantageous from an energy perspective for these bacteria to take their regulatory cues from preQ_1_ rather than its precursor. Although preQ_1_-I_I_ riboswitches regulate *queCDEF* operons and *queT* genes (74), their preference for preQ_1_ over preQ_0_ has not been documented. Based on the *Can* preQ_1_-I_I_ riboswitch structure, we expect that the β-site will not easily accommodate preQ_0_ due to a steric clash with U16 or nearby bases (Fig. 5*D*). However, it is conceivable that the riboswitch could bind mixed ligand combinations at the α/β sites, such as preQ_1_/preQ_0_ or *vice versa*. If so, these mixtures would likely lead to variations in the strength of the riboswitch’s gene-regulatory response.

## Multiple mechanisms of gene regulation by preQ_1_ riboswitch family members

To relate the atomic resolution details of preQ_1_ riboswitches to biological function, members of each riboswitch class were placed upstream of a GFP gene using an assay first described for the B_12_ riboswitch (34). PreQ_1_-dependent reporter-gene expression was then monitored using an *E. coli* Keio collection strain (121) lacking the ability to synthesize preQ_1_. The *Can* preQ_1_-I_I_ riboswitch demonstrated a strong ability to control gene expression. A plot of GFP fluorescence *versus* preQ_1_ concentration produced a biphasic curve with EC_50_ values of 80 ± 2 nM and 7.4 ± 0.5 μM. The overall change in fluorescence was 15.4 ± 1.5, indicating a strong repression ability in the presence of preQ_1_ (48). By contrast, efforts to measure the gene-regulatory activity of the *Tte* preQ_1_-I_II_ riboswitch using the same GFP assay were unsuccessful in *E. coli*. However, the *Tte* riboswitch produced a measurable dose response in *Mycobacterium smegmatis* corresponding to 2-fold overall repression. The rather weak repression could be the result of its origins from a hot spring bacterium that grows optimally at 75 °C (122).

**Figure 6:**
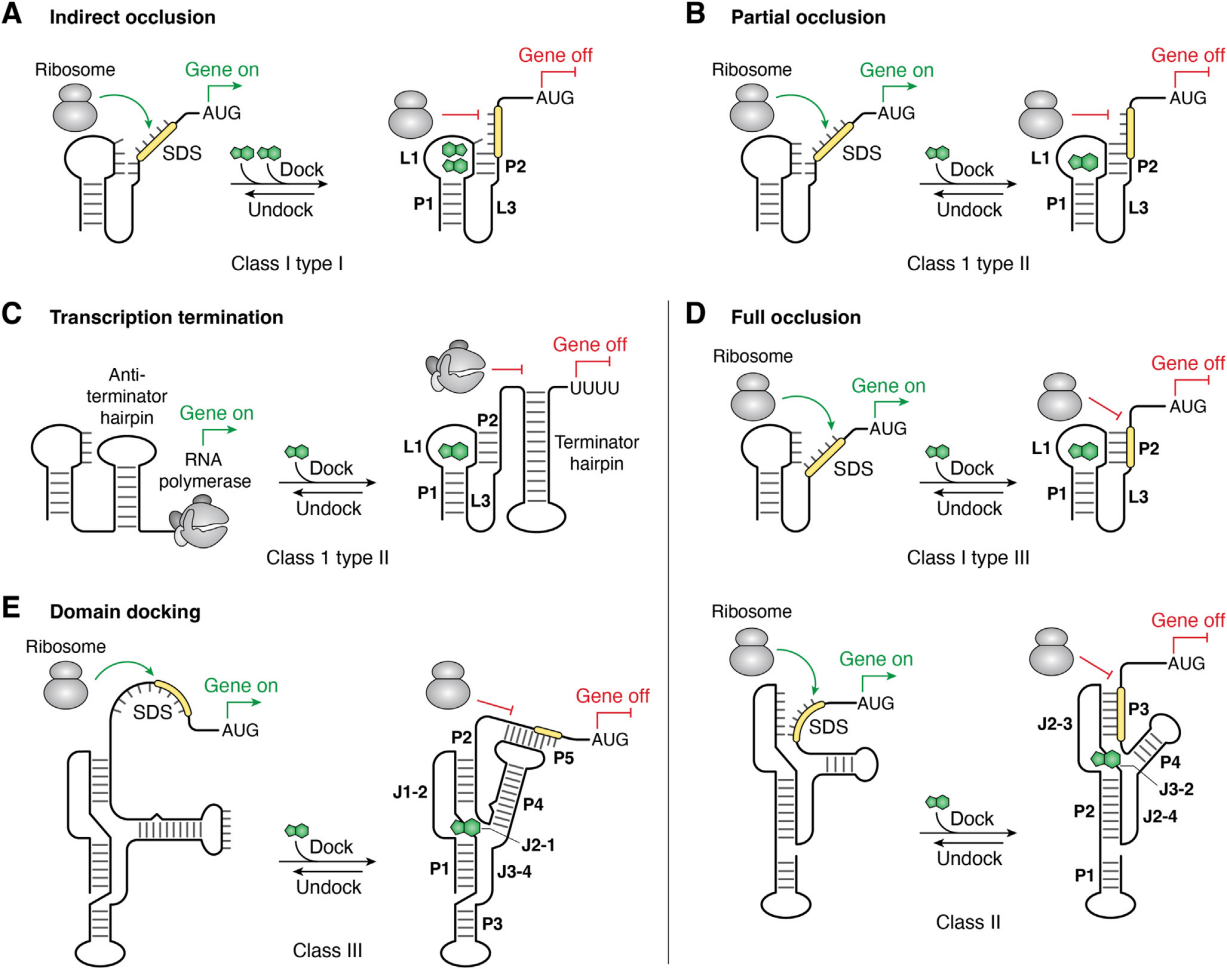
**Summary of gene-regulation strategies used by members of the preQ**_**1**_**riboswitch family.** The nomenclature adopted here is based on a recent review of riboswitch-mediated gene regulation see (128). *A*, preQ_1_-I_I_ riboswitches bind two preQ_1_ molecules that stabilize pseudoknot formation to control genes of the *queCDEF* operon and *queT* (74). Although the SDS is not strongly buried, this occlusion is sufficient to attenuate translation initiation. Notably, placement of the SDS outside the aptamer still allows gene regulation, suggesting the pseudoknot itself is more important for gene regulation than SDS sequestration (49). *B*, some preQ_1_-I_II_ riboswitches regulate *queT* genes by burial of the first two SDS nucleotides in the presence of preQ_1_, which inhibits ribosome binding. *C*, other preQ_1_-I_II_ riboswitches regulate genes of the *queCDEF* operon by use of an expression platform that controls transcription. The P2 pseudoknot helix is stabilized by preQ_1_ binding, which favors formation of an intrinsic terminator hairpin that stops the RNA polymerase from transcribing the downstream gene. *D*, the preQ_1_-I_III_ riboswitch and preQ_1_-II riboswitch regulate *yhhQ* and *queT* genes, respectively, by complete sequestration of the associated SDS upon preQ_1_ binding. These riboswitches are the quintessential examples of metabolite-sensing RNA regulators that control translation. *E*, preQ_1_-III riboswitches bind preQ_1_ using a separate domain that folds as an HL_out_ pseudoknot. The expression platform contains an SDS at the 3′-end of the riboswitch that docks with the loop of P4 to create an aSDS-SDS helix called P5. PreQ_1_ binding stimulates the population of riboswitches undergoing dynamic docking and undocking of P5, which occludes the SDS from the ribosome. PreQ_1_, prequeosine_1_; SDS, Shine-Dalgarno sequence.

A feature common to the expression platforms of translation-controlling preQ_1_-I_I_ and preQ_1_-I_II_ riboswitches is burial of the first two SDS nucleotides (Fig. 2, *A*, *B*, and *D*–*H*). Biophysical analysis of the *Tte* preQ_1_-I_II_ riboswitch indicates that the riboswitch is largely prefolded prior to preQ_1_ binding but undergoes induced fit conformational changes leading to adoption of a gene-off state that orders loop L2, reduces the overall radius of gyration, and sequesters the 5′-nt of the SDS (78, 86, 87, 88). The ability of ribosomal S1 protein to unfold the *Tte* pseudoknot depends directly on the presence of the ligand (123). Like the *Tte* preQ_1_-I_II_ riboswitch, the *Can* preQ_1_-I_I_ riboswitch was predicted to sequester only the first two SDS nucleotides. However, backbone flexibility probing by *in vivo* SHAPE showed no significant changes in SDS nucleotides A33 and G34 in the absence or presence of preQ_1_, despite overall loss of core flexibility in the ligand-bound state (49). Furthermore, molecular dynamics simulations confirmed the overall flexibility of G34, which showed paired and unpaired conformations with aSDS base C10 in various crystal structures (48, 49). Mutagenesis of G34 showed that WC base pairing is unnecessary for gene regulation, as suggested by the type I consensus model (Fig. 1*C*, *left panel*). Reverse transcription quantitative PCR showed no significant change in reporter gene transcripts at any concentration of the preQ_1_, indicating the riboswitch does not control transcription (49). Moreover, an *in vitro* RelE endonuclease assay (29) showed that the *Can* preQ_1_-I_I_ riboswitch controls translation initiation as demonstrated by progressively higher levels of translation inhibition with increasing levels of preQ_1_; the observed dose response from the RelE assay yielded a *K*_1/2_ of 16 ± 8 μΜ for preQ_1_ (49), which is slightly higher than the second EC_50_ of 7.4 ± 0.2 μΜ measured in bacterial GFP reporter assays (48). Overall, a striking aspect of the *Can* preQ_1_-I_I_ riboswitch is the ability to attenuate translation without appreciable occlusion of the SDS. Indeed, when the SDS was removed from the aptamer and separated by unstructured spacers, the *Can* riboswitch still showed significant, but reduced gene regulation up to 10 nucleotides away (49). This observation supports a weak or indirect occlusion model for gene regulation (Fig. 6*A* and Table 1). A take-home message is that the pseudoknot structure of the preQ_1_-I_I_ riboswitch appears more important for gene regulation than SDS sequestration, possibly due to the presence of two ligand binding sites. By contrast, the preQ_1_-I_II_ riboswitch appears to rely upon partial occlusion of both the first and second SDS nucleotides (Fig. 6*B* and Table 1).

**Table 1:** Representative preQ_1_ riboswitches and modes of gene regulation

Class (nomenclature)	Bacterial sequence	Pseudoknot fold	Regulation mode	Reference
Class I type I (preQ_1_-I_I_)	*Carnobacterium antarcticum* (*Can*)*, Haemophilus influenzae, Neisseria gonorrhea*	H-type	Indirect SDS Occlusion	(48, 49)
Class I type II (preQ_1_-I_II_)	*Thermoanaerobacter tengcongensis* (*Tte*)	H-type	Partial SDS Occlusion	(88)
Class I type II (preQ_1_-I_II_)	*Bacillus subtilis* (*Bsu*)	H-type	Transcription termination	(50, 51, 74)
Class I type III (preQ_1_-I_III_)	*Escherichia coli* (*Eco*)	H-type	Full SDS Occlusion	(47)
Class II (preQ_1_-II)	*Lactobacillus rhamnosus* (*Lrh*), *Streptococcus pneumoniae* (*Spn*)	HL_out_	Full SDS Occlusion	(32, 75, 76)
Class III (preQ_1_-III)	*Faecalibacterium prausnitzii* (*Fpr*), *Subdoligranulum variabile* (*Sva*)	HL_out_ (with nested H-type)	Domain Docking	(77, 111)

Preq_1,_ prequeosine_1_.

In addition to translation regulation, preQ_1_-I_II_ riboswitches, such as that from *B. subtilis*, also govern transcription (Table 1). Such control requires use of a different expression platform wherein the aptamer conformation strongly favors formation of a downstream terminator hairpin in the preQ_1_-bound state, which results in premature termination of transcription by RNA polymerase (Fig. 6*C*) (74). Interestingly, the fold of the nascent transcriptional preQ_1_ riboswitch and its interplay with the exit channel of the RNA polymerase can lead to transcriptional pausing that can be alleviated through preQ_1_ binding (51). Recently, single-particle cryo-EM captured the *Bsu* preQ_1_-I_II_ riboswitch in paused and released states in complex with RNA polymerase (50). A highly rotated, paused conformation shows retraction of the nascent RNA 3′-end from the polymerase active site. Upon binding preQ_1_, conformational changes swivel the riboswitch and enlarge the polymerase exit channel, culminating in pause release and transcription termination. This mode of RNA-based control underscores the intricate relationship between preQ_1_-I_II_ riboswitches and the transcription machinery.

Among members of the preQ_1_ class I group, the expression platform of the preQ_1_-I_III_ riboswitch stands apart because it occludes four nucleotides of the SDS (Fig. 2, *I*, *J* and *L*). In GFP reporter assays, the *Eco* riboswitch displayed a single-phase dose response to preQ_1_, which yielded an EC_50_ of 291.4 ± 1.6 μM and an overall repression level of 6.1 ± 1.7 (47). In a departure from all other members of the preQ_1_ riboswitch family, the preQ_1_-I_III_ riboswitch directly senses preQ_1_ using the first nucleotide of its SDS, A27 (Figs. 2*I* and 5*C*). The importance of A27 was probed by site-directed mutagenesis using surface plasmon resonance and *in vivo* GFP reporter assays. Mutation of A27 to purine, which lacks an exocyclic amine, weakened preQ_1_ affinity by 39,000-fold (47). To preserve the WC-base pairing properties of position 27 as well as a compatible SDS in *E. coli* (124), the A27C variant was prepared. However, no gene regulation was observed, even under saturating levels of preQ_1_ (47). Accordingly, the preQ_1_-I_III_ riboswitch appears to follow the prescribed mode of full SDS occlusion observed for other translational riboswitches, which is considered a text-book model that directly links ligand-sensing to gene regulation (Fig. 6*D* and Table 1) (7).

From an expression platform vantage point, SDS sequestration by the class II preQ_1_ riboswitch is most analogous to the preQ_1_-I_III_ riboswitch because each occludes the entire SDS in a pseudoknot helix (Figs. 2*I* and 4*B*). The class II riboswitch buries six nucleotides in aSDS-SDS P3 helix where the first SDS base, A71, forms the binding pocket floor (Fig. 4, *B*–*E*). As one would expect, the *Lrh* preQ_1_-II riboswitch produced a strong, single-phase dose response in GFP reporter assays corresponding to an EC_50_ of 19.5 ± 1.1 nM and an 11.8 ± 0.3 level of overall repression (75). The importance of preQ_1_-dependent SDS-burial is supported by chemical modification of the *Lrh* preQ_1_-II riboswitch in live bacteria caught in gene-on and off conformations using in-cell SHAPE (32, 125). Ligand-dependent changes in flexibility concur with the riboswitch crystal structure, which adopts a highly compact pseudoknot fold in the presence of preQ_1_ (*i.e.*, Fig. 4*C*) that explains the molecular basis of the gene-off state in cells (32). The importance of aSDS-SDS base pairing for gene regulation was verified by a C37G/C38G double mutant in helix P3, which was predicted to block base pairing (Fig. 4*E*). Indeed, the double mutant produced a 7600-fold loss in preQ_1_ affinity, and no gene regulation was observed (75). A C37U/C38U mutant was predicted to restore a functional aSDS–SDS interaction through tandem U•G wobbling but preQ_1_ binding was still 1100-fold worse than WT and gene regulation was abolished again (75). Single mutations of C37U or C38U were less detrimental to preQ_1_ binding—as revealed by 9- to 14-fold losses—but gene regulation was 850- to 1600-fold worser than the WT EC_50_ (75). Collectively, these experiments demonstrate that preQ_1_ affinity is not the same as biological function, and that ligand sensing by the riboswitch can be uncoupled from gene regulation. In addition, the crystal structure is an excellent representation of the gene-off state present in live bacterial cells. Hence, preQ_1_-II riboswitches exemplify a mode of control in which the entire SDS is occluded from the ribosome upon ligand binding (Fig. 6*D*)—the archetype of translation control in the riboswitch field.

Among riboswitches in the preQ_1_ family, the class III cohort stands out because its distally located expression platform controls gene expression through dynamic, ligand-stimulated interdomain docking (Fig. 6*E* and Table 1) (77). Like preQ_1_-I_II_ riboswitches, only the first two SDS nucleotides of the preQ_1_-III riboswitch are buried in aSDS-SDS P5 helix (Figs. 2, *A* and *B* and 4*K*). Recently, the preQ_1_-III riboswitch received its first validation as a gene regulator when a sequence from the gut bacterium *Subdoligranulum variabile* (*Sva*) was shown to exhibit gene-regulatory activity in bacteria using the GFP reporter assay. The single-phase dose-response curve indicated an EC_50_ of 22.5 ± 4.5 nM, which is comparable to the class II riboswitch. However, overall repression was modest at only 2.6 ± 0.5 (111). Further analysis indicated the riboswitch was more tolerant of mutations in the binding pocket wall and floor compared to the preQ_1_-II riboswitch, which shares 10 common nucleotides in its binding pocket as noted above (Fig. 5, *E* and *F*). Structures of mutant preQ_1_-III riboswitches revealed compensatory interactions that stabilized the local structure, resulting in minor defects in preQ_1_ binding (≤2-fold poorer affinity) but relatively strong retention of gene-regulatory function (4- to 90-fold poorer EC_50_ values) (111). By contrast, analogous mutations in the preQ_1_-II riboswitch were highly debilitating to preQ_1_ binding (20- to 195-fold poorer affinity) and gene regulation (7600- to 28,000-fold poorer EC_50_) (75). A take home message is that the ability of a riboswitch to accommodate mutations depends on their context in the overall fold and the ability of the riboswitch to form compensatory interactions.

## Consideration of preQ_1_ riboswitches as antibacterial targets

The ability of a riboswitch fold to accommodate mutations has implications for its suitability of as an antibiotic target. In contrast to larger preQ_1_-III riboswitches (111), smaller preQ_1_-I_III_ riboswitches are much more sensitive to core mutations possibly because 60% of all nucleotides engage in noncanonical interactions (47) (Fig. 2*I*). Although highly economical as a metabolite sensor, few possibilities exist in the preQ_1_-I_III_ aptamer to form the types of compensatory interactions observed in the preQ_1_-III riboswitch following mutagenesis (111). As a case study, we point to the large FMN riboswitch, which quickly accrued mutations inside and outside the binding pocket in the presence of the antibiotic ribocil, leading to drug resistance (54, 126). Accordingly, our observations suggest that targeting small riboswitches with compact core folds, such preQ_1_-I riboswitches, could be a better strategy for antimicrobial development (111). Other factors are also worth consideration. For example, a bioinformatic analysis of multiple riboswitches concluded that preQ_1_-I riboswitches are suitable targets due to their prevalence in multiple pathogenic bacteria, their absence in the genomes of humans and commensal bacteria, and their regulation of key biosynthetic and transporter proteins (127). Collectively, each factor—including tolerance of mutations—should be considered when sizing up a riboswitch as a potential drug target.

## Conclusions and future challenges

Riboswitches of the preQ_1_ family exhibit multiple gene-regulatory strategies (Fig. 6 and Table 1). As observed for other riboswitches, the interplay between ligand sensing and expression platform modulation is idiosyncratic with variations observed among classes and types. While some family members completely occlude the SDS (Fig. 6*D*) others partially occlude the SDS by burying the first few nucleotides (Fig. 6*B*). The most complex family member, the preQ_1_-III riboswitch, docks its distal domains to bury two SDS nucleotides in a process that is highly dynamic and stimulated by ligand binding (Fig. 6*E*). These regulatory approaches are analogous to those used by a variety of other riboswitches that control translation, which has been reviewed in a broader form elsewhere (128).

From the vantage point of transcription, the *Bsu* preQ_1_-I_II_ riboswitch functions by modulating the formation of a downstream intrinsic terminator or antiterminator hairpin that folds cotranscriptionally in response to preQ_1_ (Fig. 6*C*) (74). In the absence of ligand, the A-rich L3 loop forms part of the antiterminator hairpin (80). In the presence of preQ_1_, pseudoknot folding is favored, leading to downstream formation of an intrinsic terminator hairpin. Interestingly, the *Bsu* riboswitch also promotes elemental pausing at site immediately downstream of the riboswitch (51). Binding to preQ_1_ stimulates the release of pausing, leading to the formation of the H-type pseudoknot. Because this pausing pathway is independent of transcription factors NusA and GreB—which cause different types of pausing—it has been dubbed “pseudoknot-stabilized” pausing (51). It is hypothesized that such pausing occurs within many other bacterial transcription–elongation complexes. Notably, a riboswitch that controls transcription solely through pseudoknot-stabilized pausing would be difficult to detect by bioinformatic searches seeking to detect aptamers adjacent to an intrinsic terminator hairpin, which is dispensable in this regulatory scenario. Finding such transcriptional regulators represents a potential challenge for the field.

The regulatory prowess of the pseudoknot is also apparent in the control of translation. The preQ_1_-I_I_ riboswitch is the largest group in the preQ_1_ family based on the number of representative sequences (Fig. 1*C*, *left panel*). The type I motif stands apart because it binds two ligands in a single pocket for cooperative gene regulation (48). The latter property should confer a more digital gene-off response (*i.e.,* a regulation over a narrower ligand concentration) compared to single-ligand switch (129). An additional notable property is that ligand-dependent pseudoknot formation of the preQ_1_-I_I_ riboswitch appears more important than SDS sequestration for gene regulation (Fig. 6*A*) (49). The indirect occlusion mechanism used by the preQ_1_-I_I_ riboswitch is especially interesting when one considers that up to 35% of mRNAs are leaderless in some bacterial transcriptomes (130). In *E. coli*, approximately 30 to 50% of genes are translated without an SDS, producing a range of translation efficiencies (131). Removal of the SDS from *E. coli* transcripts showed that the corresponding mRNAs could still be translated, albeit with altered efficiency (132). These observations suggest that translation relies on other factors to control initiation. For example, the ribosomal S1 protein is necessary to dock and unfold structured messages and for proper placement of the initiation codon within the decoding channel (133). However, the S1 protein exhibited a reduced ability to unfold a preQ_1_-I_II_ riboswitch pseudoknot bound to ligand (123). The indirect occlusion model (Fig. 6*A*) also allows us to predict that riboswitches controlling translation will be found in bacteria or other organisms that do not use an SDS (49). A main implication from these findings is that the absence of an SDS diminishes the efficacy of bioinformatic searches designed to recognize riboswitches that control translation (49, 128).

How riboswitches influence the fitness of an organism in a relevant biological setting remains a poorly understood area in the riboswitch field. For example, ablation of the *tgt* gene (Fig. 1*B*) in *Shigella flexneri* compromises its ability to invade epithelial cells, suggesting a link between Q production and virulence (60). Although each member of the preQ_1_ riboswitch family has been shown to govern gene regulation under controlled conditions in cell culture (32, 47, 48, 49, 75, 82, 111), none has been tested in a true biological setting. As such, it is unknown whether mutant preQ_1_ riboswitches trapped in constitutive gene-on or gene-off states affect bacterial fitness (*e.g.,* during infection of a host cell). Establishing a link between riboswitch-mediated gene control and organism fitness remains a major challenge. Such connections will be crucial to select appropriate targets for antibiotic development. As another example, the *Vibrio cholerae* glycine riboswitch uses a tandem arrangement of homologous aptamers to sense glycine, leading to control of a single downstream expression platform (134). Biochemical and biophysical approaches led to a model for gene regulation in which both aptamers bind glycine to modulate the stability of the expression platform (97). By contrast, an *in vivo* approach in *B. subtilis* showed that mutations in the binding pocket of the second aptamer produced no effect on gene regulation (98). Moreover, mutants that constitutively express the downstream *gcvT* operon—which encodes proteins used for glycine-degradation—produced substantial defects in motility and biofilm formation under elevated concentrations of glycine, signifying a high level of selective pressure to regulate glycine (98).

Additional evidence supporting the need to analyze riboswitches in the appropriate biological context comes from comparisons of organismal fitness when mutant riboswitches are analyzed in cell culture *versus* a *S. pneumoniae* mouse–infectivity model. Mutations that confer resistance to the natural antibiotic roseoflavin disrupt ligand binding, but these variants showed no fitness advantage in cell culture in the absence of antibiotic (55). By contrast, the same constitutively gene-on mutants showed a distinct fitness disadvantage in a mouse infectivity model (55). Parallel observations were observed for constitutively gene-on mutants of the *pyrR* RNA element—a nonriboswitch motif in bacteria that controls pyrimidine biosynthesis (135). A take home message is that cell culture is not always the best way to gauge how RNA-mediated gene regulation affects organism fitness. In conclusion, our understanding of riboswitch-mediated gene regulation has advanced significantly since the discovery of the preQ_1_ riboswitch family. While the work highlighted here has exposed existing knowledge gaps, it has also identified areas of focus for the riboswitch field as a whole. We are confident that achieving these objectives will reveal exciting new modes of gene-regulation, as well as breakthroughs in antibiotic discovery and development.

## Conflict of interest

The authors declare that they have no conflicts of interest with the contents of this article.
